# Smoking can increase the risk of osteoarthritis in European women

**DOI:** 10.1038/s41598-025-09546-2

**Published:** 2025-07-03

**Authors:** Beini Mao, Hetong Li, Jintao Zhong, Xiuwang Li, Hongxun Sang

**Affiliations:** 1 Department of Orthopedics Surgery, Shenzhen Hospital, Southern Medical University, No. 13 Xinhu street, Shenzhen, 518100 Guangdong China; 2 Department of Rehabilitation Medicine, Shenzhen Hospital, Southern Medical University, No. 13 Xinhu Street, Shenzhen, 518100 China; 3 International Sports Medicine and Rehabilitation Center, Shenzhen Hospital, Southern Medical University, No. 13 Xinhu Street, Shenzhen, 518100 China; 4 Shenzhen School of Clinical Medicine, Southern Medical University, No. 13 Xinhu Street, Shenzhen, 518100 China; 5 School of Rehabilitation Sciences, Southern Medical University, Guangzhou, China

**Keywords:** Smoking, Osteoarthritis, Sex, Mendelian randomization, Etiology, Women, Osteoarthritis, Epidemiology, Outcomes research, Risk factors

## Abstract

The effect of smoking on osteoarthritis remains controversial and its effect on osteoarthritis of different genders remains unclear. This study aimed to identify the causality in different sexes and to identify the mediating effect of obesity. A two-sample mendelian randomization analysis was performed using summary data from a previous genome-wide association study and UK biobank. Analyses were performed using three methods and inverse variance weighted analysis was used as the primary method. In addition to the overall effect, the effects of smoking on osteoarthritis were evaluated separately in both sexes. The reliability of the results was evaluated using Cochran’s Q test, MR-Egger intercept test, MR PRESSO, and leave-one-out analysis. The R software was used for the analysis. There were 73 SNPs used as instrumental variables. The primary results supported that smoking led to an increased risk of osteoarthritis in both sexes (overall: OR 1.020, 95%CI 1.012–1.029; women: OR 1.024, 95%CI 1.013–1.035; men: OR 1.016, 95%CI 1.006–1.026). However, the secondary outcome did not support the effect of smoking on men (OR 0.997, 95%CI 0.949–1.047). Cochran’s Q test and MR-PRESSO test indicated that there might be some outliers in the analysis (all *p* < 0.05), while potential outliers were not found. The MR egger regression, funnel plots, and sensitivity analyses supported the robustness of the results. Mediation analysis confirmed the mediating role of obesity. Smoking increases the risk for osteoarthritis in European, especially in women, and smoking education should be recommended.

**Supplementary Information:**

The online version contains supplementary material available at 10.1038/s41598-025-09546-2.

## Background

Osteoarthritis (OA) is a common form of arthritis that primarily affects the joints. It is characterized by degeneration and breakdown of the articular cartilage as well as changes in the subchondral bone. Although most joints can be involved, OA commonly affects the knees, hips, and hands. The most common symptoms of OA include pain, stiffness, and reduced mobility^1–6^. OA is typically diagnosed through a combination of medical history, physical examination and imaging tests. Treatments of OA aim to relieve symptoms, improve joint function, and enhance quality of life. These may include lifestyle modifications, medications, assistive devices, and surgical options^7–9^. Over the past few decades, the prevalence of OA has continued to increase worldwide^10^. It was reported that the numbers are expected to continue to increase until 2050 for all OA sites, leading to a greater burden to health system^11^. Therefore, research on the modifiable risk factors that cause OA or promote its severity and disease progression is important.

There are some recognized risk factors for OA, such as obesity, knee injury, elite sports, gender, genetic susceptibility and age^12^. However, the effects of some other factors, including modifiable factors such as smoking, remain unclear on OA. Smoking is a common lifestyle factor associated with many chronic disease^13^. Previous researches suggested that smoking might be a protective factor for OA^14,15^. However, such observational studies have been weak in uncovering the causal relationships between specific factors and diseases.

Mendelian randomization (MR) is a method used in epidemiological and genetic research to investigate causal relationships between exposure and outcome^16^. It uses genetic variants known to be associated with exposure of interest as instrumental variables (IVs) to estimate the causal effect of exposure on the outcome. This approach leverages the random allocation of genetic variants during meiosis, which reduces confounding and reverse causality by mimicking the randomization process in a controlled trial. In MR analysis, genetic variants strongly associated with the exposure are selected as IVs, and their effects on the outcome are assessed using statistical methods such as inverse-variance weighting, MR-Egger regression, or weighted median approaches. This process enables the estimation of causal effects under the assumption that the IVs influence the outcome only through the exposure of interest. This method helps to overcome some limitations of observational studies, such as confounding and reverse causality^17,18^. To consistently estimate causal effects, the genetic variants used in an MR analysis must satisfy three assumptions: relevance, independence, and exclusion restrictions^19^. As MR provides a framework for investigating causal relationships between exposures and outcomes in situations where conducting randomized controlled trials may be impractical, unethical, or costly, it has become a valuable tool to infer causal relationships between exposures and outcomes, thus providing valuable insights into the potential effects of modifiable factors on human health and disease. Moreover, intermediate MR also serves to identify the mediating effects within a causal relationship^20^.

There have been a few MR studies on the effect of smoking on the incidence of OA. Some of them suggested that smoking could decrease the incidence of OA and the following total joint replacement^21,22^. However, the others suggested the opposite, believing that smoking is a risk factor for OA^23–25^. In addition to the lack of agreement in conclusions between different studies, none of the above studies considered sex as an important variable. Since sex is widely believed to play an important role in OA incidence and smoking behavior differs significantly between men and women, sex will certainly be an important confounder in exploring the impact of smoking on OA. Therefore, it is necessary to draw independent conclusions for different genders regarding the impact of smoking on OA^1,26^. Moreover, obesity, as a widely recognized risk factor for OA, has also been reported to be caused by smoking^27^. Therefore, the potential mediating role of obesity in the relationship between smoking and OA should also be validated.

Therefore, the present study aimed to identify the causality between smoking and OA, draw independent conclusions for different sexes on this issue and detect the mediating effect of obesity.

## Methods

### Study design

This study revealed the causality between smoking and OA using a two-sample MR. The data used in this study were obtained from an open source. This study did not require any ethical approval or additional informed consent owing to the use of previously collected, de-identified, and aggregated data. This work was reported in accordance with strengthening the reporting of observational epidemiological studies using Mendelian randomization (STROBE-MR)^19^. The STROBE-MR checklist is uploaded as Supplemental Material 1.

### Data sources

The largest meta-analysis of genome-wide association studies (GWASs) related to smoking was used as the exposure set. The study had a sample size of 1.2 million individuals and aimed to identify single nucleotide polymorphisms (SNPs) that were significantly associated with tobacco and alcohol^28^. The trait of smoking initiation, which indicates whether an individual had ever smoked regularly, was used to represent smoking in this study, because this trait had lower risk of bias than other traits such as amount of smoking. The sample size for this trait was 607,291 and Europeans constituted the majority of the sample.

The GWAS summary data of OA in the UK biobank was chosen as another sample and used to detect the effect of IVs on OA. In addition to the OA data, separate OA data for men and women were downloaded and analyzed. The sample sizes for overall OA, OA in women, and OA in men were 361,141, 194,153 and 166,988 respectively. Similarly, most samples were European. The detailed information on all GWAS used in this study was summarized in Supplemental Material 2.

### Selection of IVs

In the exposure set, SNPs that were strongly associated (*p* < 5 × 10^−8^) with smoking were defined as potential IVs. Linkage disequilibrium detection was performed among the extracted SNPs, with the r^2^ threshold at 0.001 and the clumping window at 10,000. If there were linkage disequilibrium, the SNPs with higher *p* values were removed. This step was completed online using the IEU website and the GWAS ID was ieu-b-4877. The R^2^ and F statistics of these SNPs were then calculated offline, and the overall F statistic was also calculated. A value of F < 10 was considered to have a weak instrument bias thus removed^29,30^. Information on such SNPs in outcome samples was also extracted by pairing chromosome number and position, whereas SNPs with a value of *p* < 5 × 10^−6^ were discarded to ensure exclusion restriction. In addition, any of the remaining SNP were searched in the GWAS catalog (https://www.ebi.ac.uk/gwas/) to determine whether they were related to any other recognized risk factors of OA and may have in horizontal pleiotropy. This includes age, knee injury history and some specific occupation^12^. If a SNP was found to be related to one of them, it would be removed. Information in both samples, such as the effect allele and other allele, was adjusted in the same direction, and the effect allele frequency was calculated by 1- minor allele frequency if the alternative allele was not a minor allele in the UK biobank data.

### Main analytical methods

The SNPs in the above procedures were used as IVs in MR analysis. The MR analysis was performed using three analytical methods. They were inverse variance weighted (IVW), MR egger, and weighted median, among which random effects IVW was set as the primary method owing to its high statistical power^19^. As for the results of the other two methods, their significances were not required while the directions of the effect sizes were used to examine the robustness of the primary results. For primary analytical method, statistical significance was set at *p* < 0.05.

### Robustness assessment

Sensitivity analyses were conducted in various ways to detect pleiotropy, which violated the main MR assumptions. Cochran’s Q test was used to detect any presence of heterogeneity across SNPs, which could indicate pleiotropy^31^. Funnel plots were also used as visualization tools to indicate horizontal pleiotropy, while asymmetry indicated the existence of pleiotropy^32^. MR-Egger regression was performed to evaluate the exclusion restriction, while the intercept represented the effect of IVs on the outcome when the effect of exposure disappeared^32^. MR PRESSO, which can identify outlier genetic variants that may bias the causal estimate, was performed to detect pleiotropy and correct the estimates by removing these outliers for horizontal pleiotropy^33^. Finally, leave-one-out analysis and its forest plot were used to evaluate the robustness of the results^34^.

### Two-step intermediate MR

A two-step MR was conducted to explore the mediate effect of obesity on this causal relationship. The largest GWAS dataset for obesity from the IEU OpenGWAS database was used to perform the analysis (ID: ukb-b-15541). In the first step, the effect (β₁) of smoking on obesity was evaluated by two sample MR. In the second step, a relaxed threshold of *p* < 5 × 10^−6^ was applied to increase instrument strength and the causal effect (β₂) of obesity on OA was evaluated. The product of the two effect estimates (β₁ × β₂) represents the mediated effect. The standard error for the indirect effect was calculated using the delta method. Sex-stratified mediation analyses were also performed to evaluate potential differences between men and women.

### Software

All MR analyses were performed using the “TwoSampleMR” package in R software^35^.

## Results

### Information on samples and IVs

There were 93 SNPs in the exposure sample met the relevance assumption and were not in linkage disequilibrium. Their information was also extracted from the outcome samples. After palindromic sequence alignment and detection of IV strength, 73 SNPs were served as the eventual IVs. Because the participants in both samples were predominantly European, the similarity of the genetic variant-exposure associations between the exposure and outcome samples was ensured. However, due to the lack of identifiable individual data, we were unable to calculate the precise overlap between the two samples. Assuming that all outcome samples were included in the exposure sample, the maximum sample overlap estimated based on the method provided by Stephen Burgess was 59%, 32%, and 27%, respectively^36^. All 93 potential SNPs and their information, including F-statistics, p value and allele frequency, are presented in Supplemental Material 3. Excluding weak IVs which were not used in the analysis, the overall F-statistic was 18.2, indicating that the IVs accurately represented the exposure.

### Main results of MR analyses

**Fig. 1:**
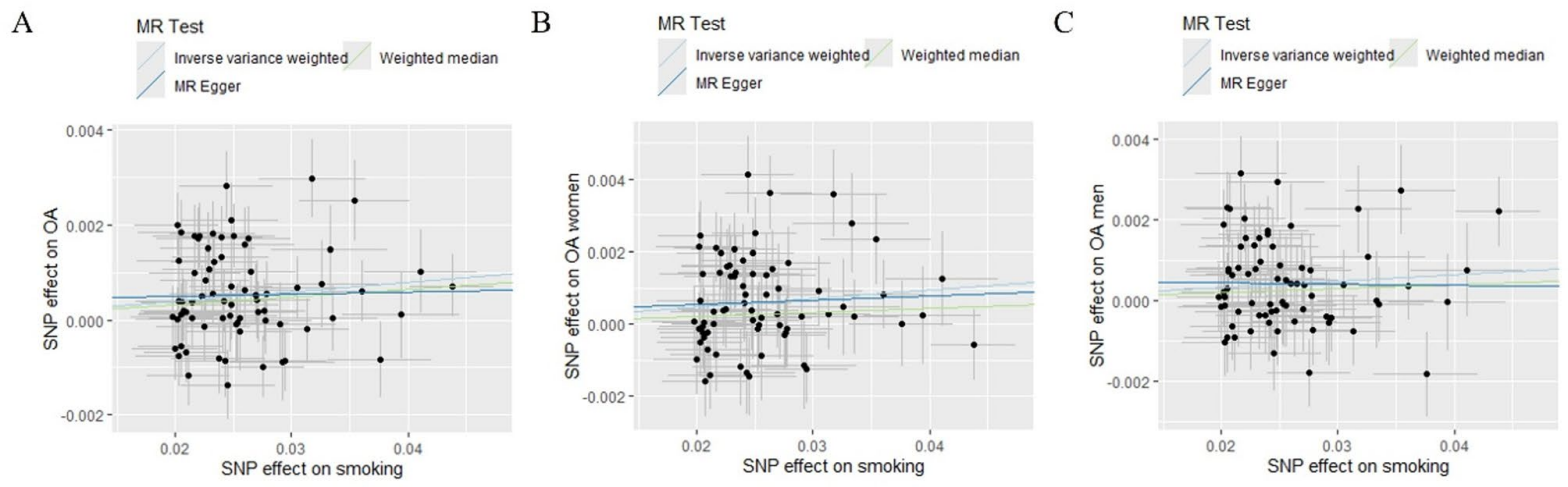
Main results of MR analysis. **(A)** The effect of smoking on overall OA. **(B)** The effect of smoking on OA in women. (C) The effect of smoking on overall OA in men.

In this two-sample MR, the primary results showed that smoking increased the risk of OA (OR 1.020, 95%CI 1.012–1.029), and this effect was evident in both sexes (Women: OR 1.024, 95%CI 1.013–1.035; Men: OR 1.016, 95%CI 1.006–1.026). All secondary results supported these results, except for the MR egger results for men (OR 0.997, 95%CI 0.949–1.047), which indicated the opposite, although this result was not significant (Fig. 1). The main results of the MR analysis using different methods are presented in Table 1.

**Table 1:** Main results of MR analysis.

Method	nSNP	β	Lower CI	Upper CI	OR	Lower CI	Upper CI	SE	*p*
*Overall*									
IVW	73	0.020	0.011	0.029	1.020	1.012	1.029	0.004	0.000
Weighted median	73	0.016	0.006	0.026	1.016	1.006	1.026	0.005	0.001
MR Egger	73	0.005	−0.039	0.048	1.005	0.962	1.049	0.022	0.835
*Female*									
IVW	73	0.024	0.013	0.035	1.024	1.013	1.035	0.006	0.000
Weighted median	73	0.011	−0.003	0.025	1.011	0.997	1.025	0.007	0.114
MR Egger	73	0.011	−0.045	0.068	1.011	0.956	1.070	0.029	0.691
*Male*									
IVW	73	0.016	0.006	0.025	1.016	1.006	1.026	0.005	0.001
Weighted median	73	0.010	−0.003	0.022	1.010	0.997	1.022	0.006	0.122
MR Egger	73	−0.003	−0.052	0.046	0.997	0.949	1.047	0.025	0.893

SNP, single-nucleotide polymorphism; OR, odds ratio; CI, confidence interval; SE, standard error; IVW, inverse-variance weighted.

### Robustness and sensitive analysis

**Fig. 2:**
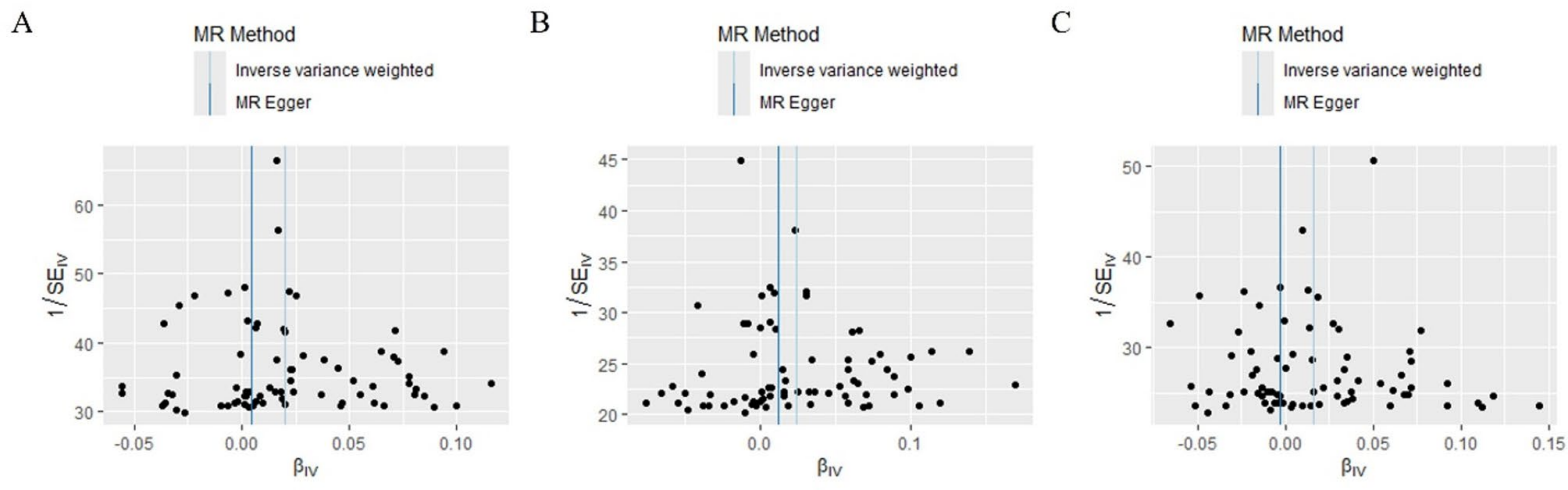
Bias detection of instrumental variables. **(A)** Instrumental variables used in the analysis of overall OA. **(B)** Instrumental variables used in the analysis of OA in women. **(C)** Instrumental variables used in the analysis of OA in men.

**Fig. 3:**
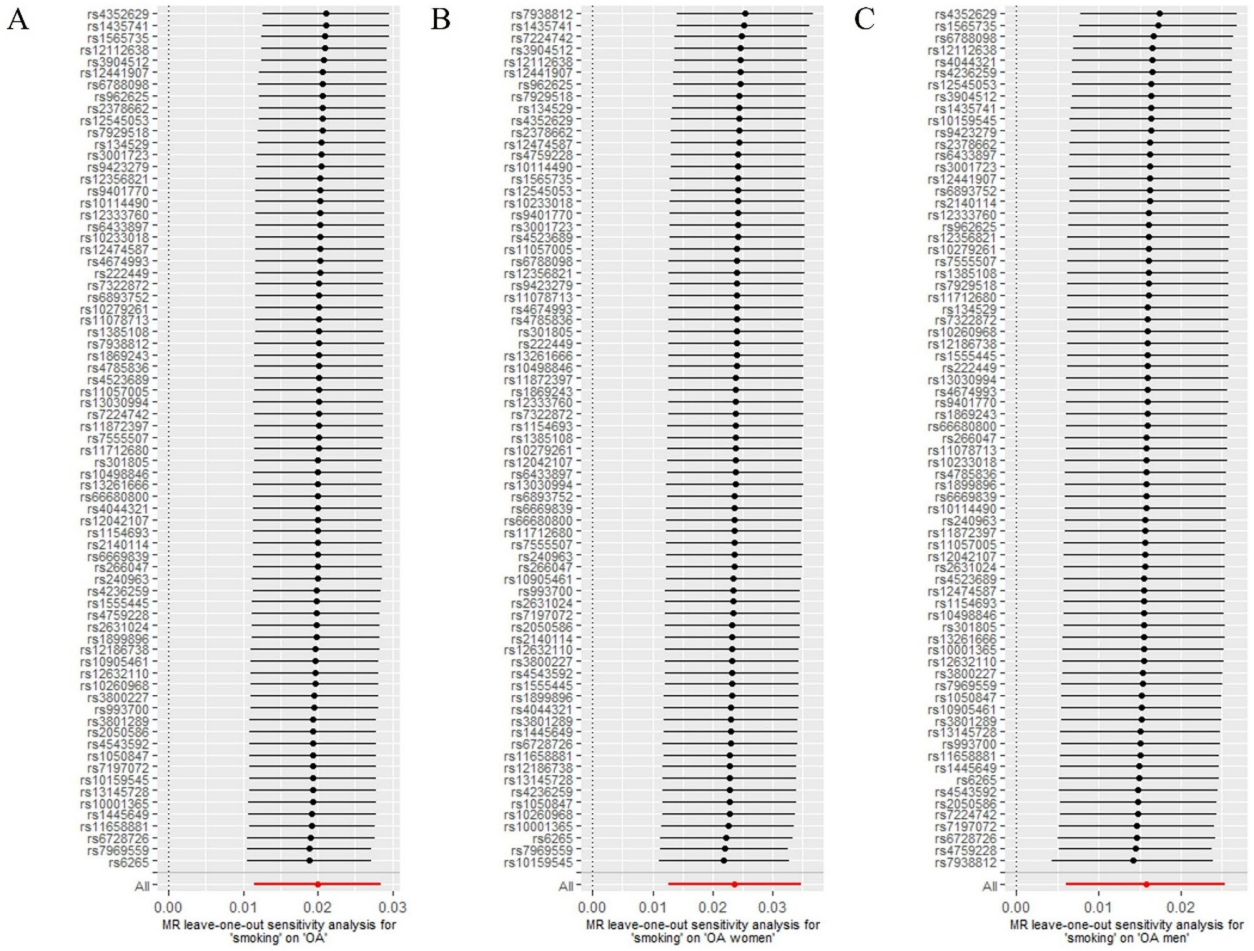
Sensitive analysis of the main results. **(A)** The effect of smoking on overall OA after removing anyone of the SNPs. **(B)** The effect of smoking on OA in women after removing anyone of the SNPs. **(C)** The effect of smoking on OA in men after removing anyone of the SNPs.

Cochran’s Q test indicated heterogeneity among the SNPs. The results are presented in Table 2. The MR-PRESSO test confirmed that there may be outliers in the analysis (all *p* < 0.05). However, potential outliers were not found in the subsequent detection even when the Nb Distribution was set to 10,000. The MR egger regression did not show any horizontal pleiotropy. The intercepts and *p* values are presented in Table 3. The funnel plots also showed no bias as the scatter points were evenly distributed on both sides of the effect size estimates (Fig. 2). Sensitivity analysis showed that the results were robust, because the removal of each SNP did not significantly change the results (Fig. 3).

**Table 2:** Results of Cochran Q test.

Group	Q	Degree of freedom	*p*
Overall	133.212	72	0.000
Female	103.450	72	0.009
Male	100.043	72	0.016

**Table 3:** Results of MR Egger regression test.

Group	Egger intercept	SE	*p*
Overall	0.000	0.001	0.478
Female	0.000	0.001	0.667
Male	0.001	0.001	0.437

### Mediation analysis

In the first step, smoking was found to be significantly associated with increased obesity (*p* < 0.001). In the second step, obesity showed a significant causal effect on OA (*p* = 0.011). When stratified by sex, obesity demonstrated a stronger and statistically significant effect on OA in women (*p* = 0.016), whereas the effect in men was positive but not statistically significant (*p* = 0.169). The results were shown in Table 4.

**Table 4:** Results of Two-Step mediation Analysis.

Causal Direction	NSNP	β	95%CI	SE	*p*
Smoking to obesity	84	0.005	(0.002,0.007)	0.001	< 0.001
Obesity to OA	13	0.606	(0.142,1.071)	0.237	0.011
Obesity to OA women	13	0.855	(0.162,1.548)	0.353	0.016
Obesity to OA men	13	0.317	(0.770,1.374)	0.231	0.169

NSNP, number of single-nucleotide polymorphism; CI, confidence interval; SE, standard error; OA, osteoarthritis.

Based on these results, the mediated effect of obesity on this causal pathway was calculated at 0.003 (95% CI: 0.000 to 0.007), accounting for 14.4% of the total effect. In women, the mediated effect was estimated at 0.004 (95% CI: 0.000 to 0.008), accounting for 16.9% of the total effect.

## Discussion

### Main findings and interpretation

This study aimed to explore whether smoking causes OA in both sexes and to explore the mediating effect of obesity. The results suggest that smoking can lead to an increased risk of OA, and that this effect is more pronounced in women. The primary results also suggested that the effect held true in men, despite the MR-Egger’s results suggested a trend toward smoking protection against OA in men. Similarly, the mediating effect of obesity was confirmed in the overall population and in women, but in men, it showed only a non-significant trend.

The overlap between samples in the main analysis might be considerable for all analyses, which might have led to weak instrumental bias. However, the F statistic demonstrated strong instrumental power, which is likely attributable to the large sample size of the selected samples. In fact, the larger the sample size is, the more negligible the effect of overlapping on results is. If the IVs are powerful, higher overlap can be ignored as well^36^. In this study, all weak IVs were removed, and the overall F statistic was 18.2. Therefore, concerns about the effect of sample overlap on reliability of the results are unnecessary.

Cochran’s Q test revealed heterogeneity among IVs, suggesting variability in the causal estimates from different SNPs. MR PRESSO confirmed the differences across IVs but failed to distinguish outliers. These results indicate the existence of horizontal pleiotropy; thus, MR-Egger regression was performed to detect it. Fortunately, no horizontal pleiotropy was detected, thus the exclusion restriction assumption was ensured. The results of this study were robust when combining forest and funnel plots.

Moreover, the results of the mediation analysis further confirmed the reliability of the primary findings and supported the mediating role of obesity in this pathway. Although mediation accounts for less than 20% suggest that most of the effect may be direct or mediated by other factors, obesity is a clear mediator, especially in women. Interestingly, just as we were unable to establish a significant causal effect of smoking on OA in men, the effect of obesity on OA in men was also not statistically significant. However, given the significance of the IVW results in the main analysis (*p* = 0.001) and the fact that both effect estimates in the two-step mediation analysis were positive, we speculate that smoking may also increase the risk of OA in men, though stronger evidence is needed to confirm this.

Multivariable MR (MVMR) was considered but was ultimately abandoned. Although MVMR offers a more robust framework for disentangling the independent effects of correlated exposures, its application remains methodologically challenging in practice. The requirement for genetic instruments across multiple exposures to be drawn from the same or highly comparable samples is often difficult to meet, especially when studying complex traits. Moreover, most publicly available GWAS summary data do not report the covariance between SNP-exposure associations, which is necessary for valid inference in MVMR^37^. These constraints are not unique to our study, but reflect broader limitations faced in the field when applying multivariable MR with summary-level data. Thus, while we opted for a univariable two-sample MR approach to preserve statistical power and instrument validity, we acknowledge that the inability to implement MVMR remains a methodological trade-off.

### Comparison with existing studies

The understanding of the relationship between smoking and OA is constantly evolving. Smoking has been thought to have a certain protective effect against OA. Most of these studies compared the incidences of OA between smokers and non-smokers or the proportion of smokers in OA and non-OA populations, and concluded that smoking was helpful in decreasing the incidence of OA based on the higher rates of OA in smokers^38–40^. A meta-analysis on this topic also supported the inverse association between cigarette smoking and risk of knee OA, suggesting a protective effect of smoking on OA^41^. Moreover, basic study indicated that the underlying mechanism might be that nicotine could reduce the cartilage degradation in OA^42^although results from animal experiments failed to confirm the results^43^. However, recent research began to realize this perspective might be wrong because of many confounding factors. An epidemiological study using a sex-specific OA risk model showed a nearly significant positive association (95% CI: 0.99–1.43) between smoking and OA in women, although the authors did not conclude that smoking was a risk factor for OA in women^44^. There were also some MR studies in recent years, which were more effective in revealing causality. One study reported that smoking behavior was causally associated with a reduced risk of OA. This result contradicts our results. In this study, an outdated GWAS database was chosen as the exposure, and only four SNPs were extracted as IVs^22^. This may have led to bias in the results. The other two studies reported similar results to ours. They pointed out that smoking had an independent deleterious causal effect on the risks of OA, as well as its subtypes^23,24^. These studies focused more on types of OA or regarded OA as a mediating factor but ignored the importance of sex in the onset of OA. The present study further investigated the impact of smoking on OA in different sexes and found that the adverse effects of smoking on OA existed in both sexes and were more significant in women.

### Potential mechanism

The different effects of smoking on OA in different sexes may be explained by the following reasons. First, it could be assumed that women might be more sensitive to smoking because in many diseases, the risks caused by smoking are greater for women than for men, after equating for tobacco exposure^45^. Second, men were reported to be more likely to quit smoking than women^46^. Considering that the IVs used in this study represented smoking initiation, which meant ever smoked, more men who quit smoking were also identified as effective compared to women, which would obviously reduce the effect size on men. Third, smoking behavior can lead to premature menopause, and subsequent estrogen deficiency can lead to degeneration of the articular cartilage, resulting in OA^47,48^. However, this mechanism is absent in men. Fourth, the mediating effect of obesity on the relationship between smoking and OA appears to be more prominent in women. Although a previous cohort study suggested that BMI played no role in the association between smoking and cartilage loss^49^our findings indicate that, in women, smoking may lead to OA through increased obesity, while this effect was not significant in men.

Identifying mediators in causal relationships plays a crucial role in disease prevention and treatment. This study confirmed the mediating role of obesity in the causal pathway from smoking to OA. However, it is noteworthy that the mediated effect (β = 0.003) accounted for only a portion of the total effect (β = 0.020). Even in the more significantly affected female population, the mediated effect was only 0.004. This suggests that the direct effect of smoking on OA is relatively large, or that other unexamined mediators may also exist.

Obesity as a mediator in the smoking–OA pathway is biologically plausible and supported by several mechanistic hypotheses. Nicotine and other components of cigarette smoke may contribute to weight gain and central adiposity by altering metabolic rate, promoting insulin resistance, and dysregulating appetite-regulating hormones such as leptin and ghrelin^50^. These changes may promote obesity-driven systemic inflammation, which is a well-established contributor to cartilage degradation and OA progression.

Interestingly, the mediation effect of obesity was observed only in the female subgroup, while this is also explainable. Postmenopausal women accumulate more fat in the intra-abdominal depot than do pre-menopausal women, and smoking increases the risk of early natural menopause, while this pathway is absent in men^48,51^.

There were also many studies that revealed disease mechanisms and identified certain proteins in disease progression, thus predicted therapeutic target^52,53^. Therefore, integrating proteomics analysis should be considered in the mediation analysis in future studies.

### Strength

Smoking has many negative effects on health^54–56^. Thus, no recommendations have been made, even though it was thought to be a potential protective factor against OA. However, the findings of the present study still have clinical implications. First, the results of this study add to the existing knowledge and confirm that smoking should be regarded as a risk factor but not a protective factor for OA. Some studies have encouraged further investigation into the effects of nicotinic receptors on joints^38^. Based on the conclusions of this study, this effort is futile. Second, the study provides the highest level of evidence on the causality of the negative effect of smoking on OA in European women. This conclusion provides a modifiable factor for the prevention of OA in certain populations who are more susceptible to OA.

### Limitation

This study has some limitations. First, both samples used in this study were based on European populations while variations in allele frequencies, linkage disequilibrium patterns, and environmental factors might be different among different populations. Therefore, the conclusions of the present study are applicable to European populations only, even though smoking and OA are common across all populations. Future studies should validate these findings in diverse ancestral populations to enhance the generalizability of the results. Second, this study focused more on sex and thus failed to provide any recommendations according to OA site. Third, we were unable to calculate the exact sample overlap due to lack of identifiable individual data, but this would not lead to much bias because of powerful IVs. Fourth, MVMR analysis was not performed. Although MVMR could help adjust for potential confounding exposures, its application requires genetic instruments for multiple exposures to be derived from the same or highly comparable samples, along with information on covariance. Given these constraints and in order to preserve instrument strength and sample quality, we opted for a univariable two-sample MR approach in this study.

## Conclusions

Smoking can lead to an increased incidence of OA in the European populations, and this effect is mainly observed in women. Smoking management should be regarded as a method for preventing OA.

## Electronic supplementary material

Below is the link to the electronic supplementary material.

Supplementary Material 1

Supplementary Material 2

Supplementary Material 3

## Data Availability

All data used were obtained from an open source and are clearly described. This information is also available from the authors Mao through polite requests.

## Abbreviations

OA: Osteoarthritis
MR: Mendelian randomization
IV: instrumental variable
STROBE-MR: strengthening the reporting of observational epidemiological studies using Mendelian randomization
GWAS: genome-wide association studies
SNP: single nucleotide polymorphism

## References

[1] Vina, E. R. & Kwoh, C. K. Epidemiology of osteoarthritis: literature update. *Curr. Opin. Rheumatol.* **30**, 160–167. 10.1097/bor.0000000000000479 (2018). 29227353 10.1097/BOR.0000000000000479 PMC5832048

[2] Prieto-Alhambra, D. et al. Incidence and risk factors for clinically diagnosed knee, hip and hand osteoarthritis: influences of age, gender and osteoarthritis affecting other joints. *Ann. Rheum. Dis.* **73**, 1659–1664. 10.1136/annrheumdis-2013-203355 (2014). 23744977 10.1136/annrheumdis-2013-203355 PMC3875433

[3] Dieppe, P. A. & Lohmander, L. S. Pathogenesis and management of pain in osteoarthritis. *Lancet (London England)*. **365**, 965–973. 10.1016/s0140-6736(05)71086-2 (2005). 15766999 10.1016/S0140-6736(05)71086-2

[4] Briggs, A. M. et al. Musculoskeletal health conditions represent a global threat to healthy aging: A report for the 2015 world health organization world report on ageing and health. *Gerontologist* **56** (Suppl 2), S243–255. 10.1093/geront/gnw002 (2016). 26994264 10.1093/geront/gnw002

[5] Turkiewicz, A. et al. Current and future impact of osteoarthritis on health care: a population-based study with projections to year 2032. *Osteoarthr. Cartil.* **22**, 1826–1832. 10.1016/j.joca.2014.07.015 (2014). 10.1016/j.joca.2014.07.015 25084132

[6] Neogi, T. The epidemiology and impact of pain in osteoarthritis. *Osteoarthr. Cartil.* **21**, 1145–1153. 10.1016/j.joca.2013.03.018 (2013). 10.1016/j.joca.2013.03.018 PMC3753584 23973124

[7] Allen, K. D., Thoma, L. M. & Golightly, Y. M. Epidemiology of osteoarthritis. *Osteoarthr. Cartil.* **30**, 184–195. 10.1016/j.joca.2021.04.020 (2022). 10.1016/j.joca.2021.04.020 PMC10735233 34534661

[8] Weber, A. E., Bolia, I. K. & Trasolini, N. A. Biological strategies for osteoarthritis: from early diagnosis to treatment. *Int. Orthop.* **45**, 335–344. 10.1007/s00264-020-04838-w (2021). 33078204 10.1007/s00264-020-04838-w

[9] Braun, H. J. & Gold, G. E. Diagnosis of osteoarthritis: imaging. *Bone* **51**, 278–288. 10.1016/j.bone.2011.11.019 (2012). 22155587 10.1016/j.bone.2011.11.019 PMC3306456

[10] Litwic, A., Edwards, M. H., Dennison, E. M. & Cooper, C. Epidemiology and burden of osteoarthritis. *Br. Med. Bull.* **105**, 185–199. 10.1093/bmb/lds038 (2013). 23337796 10.1093/bmb/lds038 PMC3690438

[11] Global & national burden of osteoarthritis. 1990–2020 and projections to 2050: a systematic analysis for the global burden of disease study 2021. *Lancet Rheumatol.* **5**, e508–e522. 10.1016/s2665-9913(23)00163-7 (2023). 37675071 10.1016/S2665-9913(23)00163-7 PMC10477960

[12] Tang, S. et al. Osteoarthritis. *Nat. Reviews Disease Primers* **11**, 10, doi:10.1038/s41572-025-00594-6 (2025). 10.1038/s41572-025-00594-6 39948092

[13] Wasfi, R. A. et al. Chronic health effects associated with electronic cigarette use: A systematic review. *Front. Public. Health*. **10**, 959622. 10.3389/fpubh.2022.959622 (2022). 36276349 10.3389/fpubh.2022.959622 PMC9584749

[14] Felson, D. T. et al. Risk factors for incident radiographic knee osteoarthritis in the elderly: the Framingham study. *Arthritis Rheum.* **40**, 728–733. 10.1002/art.1780400420 (1997). 9125257 10.1002/art.1780400420

[15] Racunica, T. L. et al. A positive association of smoking and articular knee joint cartilage in healthy people. *Osteoarthr. Cartil.* **15**, 587–590. 10.1016/j.joca.2006.12.005 (2007). 10.1016/j.joca.2006.12.005 17291790

[16] Emdin, C. A., Khera, A. V. & Kathiresan, S. *Mendelian Randomization Jama* **318**, 1925–1926, doi:10.1001/jama.2017.17219 (2017). 29164242 10.1001/jama.2017.17219

[17] Bowden, J. & Holmes, M. V. Meta-analysis and Mendelian randomization: A review. *Res. Synthesis Methods*. **10**, 486–496. 10.1002/jrsm.1346 (2019). 10.1002/jrsm.1346 PMC6973275 30861319

[18] Birney, E. Mendelian randomization. *Cold Spring Harbor Perspect. Med.* **12**10.1101/cshperspect.a041302 (2022). 10.1101/cshperspect.a041302 PMC9121891 34872952

[19] Skrivankova, V. W. et al. Strengthening the reporting of observational studies in epidemiology using Mendelian randomisation (STROBE-MR): explanation and elaboration. *Bmj* **375**, n2233. 10.1136/bmj.n2233 (2021). 34702754 10.1136/bmj.n2233 PMC8546498

[20] Carter, A. R. et al. Mendelian randomisation for mediation analysis: current methods and challenges for implementation. *Eur. J. Epidemiol.* **36**, 465–478. 10.1007/s10654-021-00757-1 (2021). 33961203 10.1007/s10654-021-00757-1 PMC8159796

[21] Johnsen, M. B. et al. The causal role of smoking on the risk of hip or knee replacement due to primary osteoarthritis: a Mendelian randomisation analysis of the HUNT study. *Osteoarthr. Cartil.* **25**, 817–823. 10.1016/j.joca.2016.12.021 (2017). 10.1016/j.joca.2016.12.021 28049019

[22] Lee, Y. H. Causal association between smoking behavior and the decreased risk of osteoarthritis: a Mendelian randomization. *Z. Rheumatol.* **78**, 461–466. 10.1007/s00393-018-0505-7 (2019). 29974223 10.1007/s00393-018-0505-7

[23] Gill, D., Karhunen, V., Malik, R., Dichgans, M. & Sofat, N. Cardiometabolic traits mediating the effect of education on osteoarthritis risk: a Mendelian randomization study. *Osteoarthr. Cartil.* **29**, 365–371. 10.1016/j.joca.2020.12.015 (2021). 10.1016/j.joca.2020.12.015 PMC7955282 33422704

[24] Ni, J. et al. Does smoking protect against developing osteoarthritis? Evidence from a genetically informed perspective. *Semin. Arthritis Rheum.* **55**, 152013. 10.1016/j.semarthrit.2022.152013 (2022). 35500427 10.1016/j.semarthrit.2022.152013

[25] Larsson, S. C. & Burgess, S. Appraising the causal role of smoking in multiple diseases: A systematic review and meta-analysis of Mendelian randomization studies. *EBioMedicine* **82**, 104154. 10.1016/j.ebiom.2022.104154 (2022). 35816897 10.1016/j.ebiom.2022.104154 PMC9278068

[26] Teixeira-da-Costa, E. M., Merino-Godoy, M. D., Almeida, M., Silva, A. & Nave, F. Gender and tobacco consumption among university students. *Int. J. Environ. Res. Public Health*. **19**10.3390/ijerph192214772 (2022). 10.3390/ijerph192214772 PMC9690291 36429490

[27] Park, S. et al. Causal effects from tobacco smoking initiation on obesity-related traits: a Mendelian randomization study. *International journal of obesity (*) 47, 1232–1238,) 47, 1232–1238, (2005). 10.1038/s41366-023-01371-9 (2023). 10.1038/s41366-023-01371-9 37634025

[28] Liu, M. et al. Association studies of up to 1.2 million individuals yield new insights into the genetic etiology of tobacco and alcohol use. *Nat. Genet.* **51**, 237–244. 10.1038/s41588-018-0307-5 (2019). 30643251 10.1038/s41588-018-0307-5 PMC6358542

[29] Palmer, T. M. et al. Using multiple genetic variants as instrumental variables for modifiable risk factors. *Stat. Methods Med. Res.* **21**, 223–242. 10.1177/0962280210394459 (2012). 21216802 10.1177/0962280210394459 PMC3917707

[30] Burgess, S. & Thompson, S. G. Avoiding bias from weak instruments in Mendelian randomization studies. *Int. J. Epidemiol.* **40**, 755–764. 10.1093/ije/dyr036 (2011). 21414999 10.1093/ije/dyr036

[31] Cole, S. R. & Frangakis, C. E. The consistency statement in causal inference: a definition or an assumption? *Epidemiol. (Cambridge Mass)*. **20**, 3–5. 10.1097/EDE.0b013e31818ef366 (2009). 10.1097/EDE.0b013e31818ef366 19234395

[32] Bowden, J., Davey Smith, G. & Burgess, S. Mendelian randomization with invalid instruments: effect Estimation and bias detection through Egger regression. *Int. J. Epidemiol.* **44**, 512–525. 10.1093/ije/dyv080 (2015). 26050253 10.1093/ije/dyv080 PMC4469799

[33] Verbanck, M., Chen, C. Y., Neale, B. & Do, R. Detection of widespread horizontal Pleiotropy in causal relationships inferred from Mendelian randomization between complex traits and diseases. *Nat. Genet.* **50**, 693–698. 10.1038/s41588-018-0099-7 (2018). 29686387 10.1038/s41588-018-0099-7 PMC6083837

[34] Hemani, G. et al. The MR-Base platform supports systematic causal inference across the human phenome. *eLife* **7**10.7554/eLife.34408 (2018). 10.7554/eLife.34408 PMC5976434 29846171

[35] Hemani, G. & Tilling, K. Davey smith, G. Orienting the causal relationship between imprecisely measured traits using GWAS summary data. *PLoS Genet.* **13**, e1007081. 10.1371/journal.pgen.1007081 (2017). 29149188 10.1371/journal.pgen.1007081 PMC5711033

[36] Burgess, S., Davies, N. M. & Thompson, S. G. Bias due to participant overlap in two-sample Mendelian randomization. *Genet. Epidemiol.* **40**, 597–608. 10.1002/gepi.21998 (2016). 27625185 10.1002/gepi.21998 PMC5082560

[37] Zuber, V., Colijn, J. M., Klaver, C. & Burgess, S. Selecting likely causal risk factors from high-throughput experiments using multivariable Mendelian randomization. *Nat. Commun.* **11**, 29. 10.1038/s41467-019-13870-3 (2020). 31911605 10.1038/s41467-019-13870-3 PMC6946691

[38] Roux, C. H. et al. Impact of smoking on femorotibial and hip osteoarthritis progression: 3-year follow-up data from the KHOALA cohort. *Joint Bone Spine*. **88**, 105077. 10.1016/j.jbspin.2020.09.009 (2021). 32950705 10.1016/j.jbspin.2020.09.009

[39] Mnatzaganian, G., Ryan, P., Norman, P. E., Davidson, D. C. & Hiller, J. E. Smoking, body weight, physical exercise, and risk of lower limb total joint replacement in a population-based cohort of men. *Arthritis Rheum.* **63**, 2523–2530. 10.1002/art.30400 (2011). 21748729 10.1002/art.30400

[40] Järvholm, B., Lewold, S., Malchau, H. & Vingård, E. Age, bodyweight, smoking habits and the risk of severe osteoarthritis in the hip and knee in men. *Eur. J. Epidemiol.* **20**, 537–542. 10.1007/s10654-005-4263-x (2005). 16121763 10.1007/s10654-005-4263-x

[41] Kong, L., Wang, L., Meng, F., Cao, J. & Shen, Y. Association between smoking and risk of knee osteoarthritis: a systematic review and meta-analysis. *Osteoarthr. Cartil.* **25**, 809–816. 10.1016/j.joca.2016.12.020 (2017). 10.1016/j.joca.2016.12.020 28011100

[42] Teng, P. et al. Nicotine Attenuates Osteoarthritis Pain and Matrix Metalloproteinase-9 Expression via the α7 Nicotinic Acetylcholine Receptor. *Journal of immunology (Baltimore, Md.*:) 203, 485–492, ) 203, 485–492, (1950). 10.4049/jimmunol.1801513 (2019). 10.4049/jimmunol.1801513 31152077

[43] Bock, K. et al. What is the effect of nicotinic acetylcholine receptor stimulation on osteoarthritis in a rodent animal model? *SAGE Open. Med.* **4**10.1177/2050312116637529 (2016). 10.1177/2050312116637529 PMC4790423 27026802

[44] Szilagyi, I. A., Waarsing, J. H., Schiphof, D., van Meurs, J. B. J. & Bierma-Zeinstra, S. M. A. Towards sex-specific osteoarthritis risk models: evaluation of risk factors for knee osteoarthritis in males and females. *Rheumatol. (Oxford)*. **61**, 648–657. 10.1093/rheumatology/keab378 (2022). 10.1093/rheumatology/keab378 PMC8824415 33895803

[45] McKee, S. A. & McRae-Clark, A. L. Consideration of sex and gender differences in addiction medication response. *Biology Sex. Differences*. **13**10.1186/s13293-022-00441-3 (2022). 10.1186/s13293-022-00441-3 PMC9235243 35761351

[46] Smith, P. H., Bessette, A. J., Weinberger, A. H., Sheffer, C. E. & McKee, S. A. Sex/gender differences in smoking cessation: A review. *Prev. Med.* **92**, 135–140. 10.1016/j.ypmed.2016.07.013 (2016). 27471021 10.1016/j.ypmed.2016.07.013 PMC5085924

[47] Xu, X. et al. Estrogen modulates cartilage and subchondral bone remodeling in an ovariectomized rat model of postmenopausal osteoarthritis. *Med. Sci. Monitor: Int. Med. J. Experimental Clin. Res.* **25**, 3146–3153. 10.12659/msm.916254 (2019). 10.12659/MSM.916254 PMC6503753 31031401

[48] Sun, L. et al. Meta-analysis suggests that smoking is associated with an increased risk of early natural menopause. *Menopause (New York N Y)*. **19**, 126–132. 10.1097/gme.0b013e318224f9ac (2012). 21946090 10.1097/gme.0b013e318224f9ac

[49] Shen, Z., Wang, Y., Xing, X., Jones, G. & Cai, G. Association of smoking with cartilage loss of knee osteoarthritis: data from two longitudinal cohorts. *BMC Musculoskelet. Disord*. **24**, 812. 10.1186/s12891-023-06953-2 (2023). 37833699 10.1186/s12891-023-06953-2 PMC10571432

[50] Chiolero, A., Faeh, D., Paccaud, F. & Cornuz, J. Consequences of smoking for body weight, body fat distribution, and insulin resistance. *Am. J. Clin. Nutr.* **87**, 801–809. 10.1093/ajcn/87.4.801 (2008). 18400700 10.1093/ajcn/87.4.801

[51] Shi, H. & Clegg, D. J. Sex differences in the regulation of body weight. *Physiol. Behav.* **97**, 199–204. 10.1016/j.physbeh.2009.02.017 (2009). 19250944 10.1016/j.physbeh.2009.02.017 PMC4507503

[52] Zhang, Y. et al. Evaluating the causal effect of Circulating proteome on the risk of osteoarthritis-related traits. *Ann. Rheum. Dis.* **82**, 1606–1617. 10.1136/ard-2023-224459 (2023). 37595989 10.1136/ard-2023-224459

[53] Xing, X. et al. Evaluating the causal effect of Circulating proteome on glycemic traits: evidence from Mendelian randomization. *Diabetes* **74**, 108–119. 10.2337/db24-0262 (2025). 39418314 10.2337/db24-0262

[54] Yuan, S. et al. Smoking, alcohol consumption, and 24 gastrointestinal diseases: Mendelian randomization analysis. *eLife* 12, (2023). 10.7554/eLife.84051 10.7554/eLife.84051 PMC10017103 36727839

[55] Mo, C. et al. Evaluating the causal effect of tobacco smoking on white matter brain aging: a two-sample Mendelian randomization analysis in UK biobank. *Addict. (Abingdon England)*. **118**, 739–749. 10.1111/add.16088 (2023). 10.1111/add.16088 PMC10443605 36401354

[56] Kotlyarov, S. The role of smoking in the mechanisms of development of chronic obstructive pulmonary disease and atherosclerosis. *Int. J. Mol. Sci.* **24**10.3390/ijms24108725 (2023). 10.3390/ijms24108725 PMC10217854 37240069

