# Supplementary material for: Smoking can increase the risk of osteoarthritis in European women
Source: Sci Rep. 2025 Jul 3;15:23750. doi: 10.1038/s41598-025-09546-2 (PMC12229534; doi:10.1038/s41598-025-09546-2)
Supplement: Supplementary file 2 — Supplementary Material 2 [file 41598_2025_9546_MOESM2_ESM.docx]

Supplemental material 2. Information on GWAS summary data.

| Trait | Year | Consortium | Sample sizes | Dominant population | Download link |
| --- | --- | --- | --- | --- | --- |
| Smoking | 2019 | GSCAN | 607291 | European | https://gwas.mrcieu.ac.uk/datasets/ieu-b-4877/ |
| OA | 2018 | UK biobank | 361141 | European | https://broad-ukb-sumstats-us-east-1.s3.amazonaws.com/round2/additive-tsvs/20002_1465.gwas.imputed_v3.both_sexes.tsv.bgz |
| OA women | 2018 | UK biobank | 194153 | European | https://broad-ukb-sumstats-us-east-1.s3.amazonaws.com/round2/additive-tsvs/20002_1465.gwas.imputed_v3.female.tsv.bgz |
| OA men | 2018 | UK biobank | 166988 | European | https://broad-ukb-sumstats-us-east-1.s3.amazonaws.com/round2/additive-tsvs/20002_1465.gwas.imputed_v3.male.tsv.bgz |
| Obesity | 2018 | MRC-IEU | 463010 | European | https://gwas.mrcieu.ac.uk/datasets/ukb-b-15541/ |
