# Supplementary material for: Smoking can increase the risk of osteoarthritis in European women
Source: Sci Rep. 2025 Jul 3;15:23750. doi: 10.1038/s41598-025-09546-2 (PMC12229534; doi:10.1038/s41598-025-09546-2)
Supplement: Supplementary file 3 — Supplementary Material 3 [file 41598_2025_9546_MOESM3_ESM.docx]

Supplemental Material 3. Information on Potential Instrumental Variants

| SNP | EA | BA | EAF | β | SE | *p* | R^2^ | F |
| --- | --- | --- | --- | --- | --- | --- | --- | --- |
| rs10001365 | A | G | 0.405 | -0.02499 | 0.00364 | 6.65E-12 | 3.59E-05 | 22.701 |
| rs10114490 | A | G | 0.198 | -0.02551 | 0.00453 | 1.81E-08 | 1.59E-05 | 10.068 |
| rs10159545 | G | C | 0.375 | 0.02625 | 0.00373 | 1.84E-12 | 3.67E-05 | 23.251 |
| rs10233018 | G | A | 0.503 | 0.02707 | 0.00356 | 2.75E-14 | 4.57E-05 | 28.950 |
| rs10260968 | A | G | 0.597 | -0.02032 | 0.00361 | 1.75E-08 | 2.41E-05 | 15.254 |
| rs10279261 | A | G | 0.619 | -0.02142 | 0.00366 | 5.00E-09 | 2.55E-05 | 16.132 |
| rs10498846 | T | C | 0.473 | 0.02061 | 0.00356 | 6.62E-09 | 2.65E-05 | 16.751 |
| rs1050847 | T | C | 0.505 | -0.02162 | 0.00359 | 1.67E-09 | 2.87E-05 | 18.149 |
| rs10905461 | C | T | 0.718 | -0.02396 | 0.00415 | 7.35E-09 | 2.14E-05 | 13.526 |
| rs11057005 | G | A | 0.43 | -0.02093 | 0.00358 | 4.85E-09 | 2.65E-05 | 16.765 |
| rs11078713 | G | A | 0.454 | -0.02017 | 0.00361 | 2.23E-08 | 2.45E-05 | 15.518 |
| rs1154693 | G | A | 0.856 | 0.03262 | 0.00491 | 3.12E-11 | 1.72E-05 | 10.872 |
| rs1160685 | G | C | 0.478 | 0.02077 | 0.00359 | 7.20E-09 | 2.64E-05 | 16.718 |
| rs11658881 | G | A | 0.418 | 0.02014 | 0.00361 | 2.43E-08 | 2.39E-05 | 15.132 |
| rs11712680 | C | A | 0.174 | -0.02705 | 0.00458 | 3.51E-09 | 1.59E-05 | 10.032 |
| rs117143374 | C | T | 0.12 | 0.02929 | 0.00527 | 2.76E-08 | 1.03E-05 | 6.526 |
| rs11872397 | A | G | 0.252 | -0.02477 | 0.00409 | 1.43E-09 | 2.18E-05 | 13.798 |
| rs12025237 | C | A | 0.124 | -0.033 | 0.00534 | 6.52E-10 | 1.31E-05 | 8.299 |
| rs12042107 | C | T | 0.527 | -0.02228 | 0.00357 | 4.22E-10 | 3.07E-05 | 19.444 |
| rs12112638 | G | A | 0.275 | -0.02453 | 0.00404 | 1.34E-09 | 2.32E-05 | 14.674 |
| rs12186738 | T | G | 0.154 | -0.03326 | 0.00502 | 3.42E-11 | 1.81E-05 | 11.439 |
| rs12333760 | C | T | 0.204 | -0.02905 | 0.0048 | 1.44E-09 | 1.88E-05 | 11.887 |
| rs12356821 | C | G | 0.14 | 0.03937 | 0.00505 | 6.27E-15 | 2.31E-05 | 14.641 |
| rs12441907 | A | C | 0.186 | -0.02921 | 0.00452 | 1.06E-10 | 2.00E-05 | 12.627 |
| rs12474587 | T | G | 0.404 | 0.02763 | 0.00358 | 1.25E-14 | 4.53E-05 | 28.655 |
| rs12545053 | G | A | 0.397 | 0.02028 | 0.00364 | 2.43E-08 | 2.35E-05 | 14.890 |
| rs12632110 | G | A | 0.647 | -0.02338 | 0.00375 | 4.78E-10 | 2.80E-05 | 17.724 |
| rs13030994 | A | G | 0.485 | 0.03609 | 0.00356 | 3.56E-24 | 8.13E-05 | 51.458 |
| rs13145728 | C | G | 0.358 | -0.02325 | 0.00366 | 2.14E-10 | 2.93E-05 | 18.525 |
| rs13261666 | T | G | 0.522 | -0.02689 | 0.00356 | 3.90E-14 | 4.51E-05 | 28.546 |
| rs134529 | C | T | 0.349 | -0.01998 | 0.00366 | 4.85E-08 | 2.14E-05 | 13.541 |
| rs1385108 | T | C | 0.239 | 0.02466 | 0.00416 | 3.00E-09 | 2.02E-05 | 12.805 |
| rs1435741 | A | G | 0.425 | 0.02942 | 0.00359 | 2.64E-16 | 5.18E-05 | 32.797 |
| rs1445649 | C | T | 0.525 | 0.02399 | 0.00356 | 1.68E-11 | 3.57E-05 | 22.594 |
| rs1555445 | T | A | 0.337 | 0.02255 | 0.00382 | 3.65E-09 | 2.46E-05 | 15.551 |
| rs1565735 | A | T | 0.212 | -0.03762 | 0.00446 | 3.42E-17 | 3.75E-05 | 23.756 |
| rs1869243 | C | T | 0.481 | 0.01974 | 0.00356 | 2.97E-08 | 2.42E-05 | 15.328 |
| rs1899896 | T | C | 0.286 | 0.02645 | 0.00389 | 1.04E-11 | 2.99E-05 | 18.910 |
| rs1971318 | T | C | 0.141 | 0.02851 | 0.00493 | 7.06E-09 | 1.28E-05 | 8.115 |
| rs2046850 | T | C | 0.187 | -0.02481 | 0.00448 | 3.03E-08 | 1.48E-05 | 9.335 |
| rs2050586 | C | G | 0.355 | -0.02055 | 0.00371 | 3.00E-08 | 2.22E-05 | 14.059 |
| rs2107300 | G | C | 0.845 | -0.0272 | 0.00493 | 3.27E-08 | 1.26E-05 | 7.990 |
| rs2140114 | T | C | 0.518 | -0.02326 | 0.00373 | 4.70E-10 | 3.06E-05 | 19.375 |
| rs2186122 | T | A | 0.561 | 0.02606 | 0.00359 | 3.61E-13 | 4.11E-05 | 26.008 |
| rs222449 | T | A | 0.793 | -0.02532 | 0.00443 | 1.08E-08 | 1.70E-05 | 10.736 |
| rs2378662 | A | G | 0.556 | 0.02095 | 0.00357 | 4.16E-09 | 2.69E-05 | 17.034 |
| rs240963 | C | T | 0.836 | -0.04104 | 0.00484 | 2.16E-17 | 3.12E-05 | 19.744 |
| rs2631024 | G | A | 0.737 | -0.02296 | 0.00403 | 1.18E-08 | 1.99E-05 | 12.599 |
| rs266047 | A | G | 0.529 | -0.03051 | 0.00374 | 3.36E-16 | 5.24E-05 | 33.190 |
| rs3001723 | A | G | 0.321 | 0.03351 | 0.0039 | 8.12E-18 | 5.09E-05 | 32.216 |
| rs301805 | G | T | 0.559 | 0.02147 | 0.00361 | 2.80E-09 | 2.75E-05 | 17.405 |
| rs35702515 | T | G | 0.162 | 0.02524 | 0.00423 | 2.43E-09 | 1.53E-05 | 9.666 |
| rs3800227 | G | A | 0.701 | 0.02281 | 0.00406 | 1.93E-08 | 2.09E-05 | 13.247 |
| rs3801289 | C | A | 0.351 | -0.02206 | 0.00374 | 3.74E-09 | 2.51E-05 | 15.855 |
| rs3904512 | A | G | 0.429 | -0.02116 | 0.00358 | 3.23E-09 | 2.71E-05 | 17.148 |
| rs4044321 | G | A | 0.642 | -0.02784 | 0.00371 | 6.08E-14 | 4.09E-05 | 25.881 |
| rs4236259 | G | T | 0.499 | -0.02477 | 0.00356 | 3.35E-12 | 3.83E-05 | 24.251 |
| rs4352629 | T | C | 0.492 | -0.02753 | 0.00357 | 1.22E-14 | 4.70E-05 | 29.744 |
| rs4523689 | G | A | 0.408 | -0.02061 | 0.00364 | 1.55E-08 | 2.44E-05 | 15.459 |
| rs4543592 | C | T | 0.468 | 0.02193 | 0.00356 | 7.46E-10 | 2.98E-05 | 18.873 |
| rs4674993 | G | A | 0.207 | -0.02521 | 0.00444 | 1.32E-08 | 1.68E-05 | 10.604 |
| rs4759228 | C | G | 0.27 | -0.02169 | 0.00393 | 3.58E-08 | 1.89E-05 | 11.984 |
| rs4781977 | C | T | 0.205 | -0.02387 | 0.00436 | 4.54E-08 | 1.54E-05 | 9.746 |
| rs4785836 | C | T | 0.398 | -0.02047 | 0.00366 | 2.26E-08 | 2.37E-05 | 14.999 |
| rs578584 | T | A | 0.605 | 0.02868 | 0.0036 | 1.50E-15 | 4.80E-05 | 30.399 |
| rs6265 | T | C | 0.203 | -0.03179 | 0.00458 | 3.77E-12 | 2.46E-05 | 15.597 |
| rs6433897 | C | T | 0.754 | 0.02245 | 0.00406 | 3.16E-08 | 1.79E-05 | 11.352 |
| rs6508144 | G | C | 0.563 | -0.02069 | 0.00359 | 7.97E-09 | 2.59E-05 | 16.386 |
| rs66680800 | T | G | 0.397 | -0.02027 | 0.00365 | 2.83E-08 | 2.33E-05 | 14.747 |
| rs6669839 | T | C | 0.204 | 0.026 | 0.0044 | 3.36E-09 | 1.80E-05 | 11.367 |
| rs6728726 | C | T | 0.829 | 0.03545 | 0.00473 | 6.73E-14 | 2.51E-05 | 15.906 |
| rs6788098 | T | A | 0.623 | -0.03135 | 0.00369 | 1.91E-17 | 5.36E-05 | 33.917 |
| rs6893752 | G | A | 0.766 | -0.0241 | 0.00407 | 3.25E-09 | 1.98E-05 | 12.547 |
| rs7197072 | T | C | 0.238 | -0.02477 | 0.00417 | 2.77E-09 | 2.02E-05 | 12.804 |
| rs7224742 | T | C | 0.595 | -0.02071 | 0.00366 | 1.43E-08 | 2.44E-05 | 15.471 |
| rs72789632 | T | C | 0.12 | -0.03289 | 0.00529 | 5.02E-10 | 1.29E-05 | 8.174 |
| rs72896886 | C | G | 0.144 | -0.02689 | 0.00484 | 2.75E-08 | 1.20E-05 | 7.618 |
| rs7322872 | T | C | 0.782 | -0.02557 | 0.00433 | 3.58E-09 | 1.88E-05 | 11.865 |
| rs7555507 | T | C | 0.496 | -0.02414 | 0.00356 | 1.14E-11 | 3.64E-05 | 23.049 |
| rs7585579 | G | C | 0.505 | 0.0224 | 0.00373 | 1.88E-09 | 2.85E-05 | 18.049 |
| rs76214862 | C | A | 0.202 | -0.02499 | 0.00455 | 3.99E-08 | 1.54E-05 | 9.736 |
| rs76608582 | A | C | 0.0389 | -0.04956 | 0.00826 | 1.94E-09 | 4.25E-06 | 2.692 |
| rs78411160 | C | A | 0.631 | 0.02054 | 0.00366 | 2.03E-08 | 2.32E-05 | 14.669 |
| rs7921378 | C | G | 0.463 | -0.02546 | 0.00356 | 8.26E-13 | 4.02E-05 | 25.461 |
| rs7929518 | G | A | 0.765 | 0.02424 | 0.00428 | 1.56E-08 | 1.82E-05 | 11.506 |
| rs7938812 | G | T | 0.424 | 0.04379 | 0.00364 | 2.71E-33 | 0.000112 | 70.833 |
| rs7969559 | G | A | 0.688 | -0.02438 | 0.00396 | 7.31E-10 | 2.57E-05 | 16.271 |
| rs9401770 | A | G | 0.273 | 0.02773 | 0.00399 | 3.47E-12 | 3.04E-05 | 19.213 |
| rs9423279 | G | C | 0.641 | -0.02051 | 0.00371 | 3.21E-08 | 2.23E-05 | 14.084 |
| rs9540729 | T | A | 0.501 | -0.01955 | 0.00356 | 3.82E-08 | 2.39E-05 | 15.100 |
| rs962625 | G | A | 0.24 | 0.02372 | 0.00404 | 4.37E-09 | 1.99E-05 | 12.586 |
| rs9835772 | T | A | 0.235 | 0.02405 | 0.00414 | 6.32E-09 | 1.91E-05 | 12.117 |
| rs993700 | C | T | 0.766 | -0.02593 | 0.00429 | 1.53E-09 | 2.07E-05 | 13.085 |

SNP, Single-nucleotide polymorphism; EA, Effect allele; BA, Baseline allele; EAF, Effect allele frequency; SE, Standard error.
